# Effective xanthine oxidase inhibitor urate lowering therapy in gout is linked to an emergent serum protein interactome of complement and inflammation modulators

**DOI:** 10.1038/s41598-024-74154-5

**Published:** 2024-10-19

**Authors:** Concepcion Sanchez, Anaamika Campeau, Ru Liu-Bryan, Ted R. Mikuls, James R. O’Dell, David J. Gonzalez, Robert Terkeltaub

**Affiliations:** 1 Department of Pharmacology, University of California, San Diego, CA USA; 2 Skaggs School of Pharmacy and Pharmaceutical Sciences, and Collaborative Center for Multiplexed Proteomics, University of California, San Diego, La Jolla, CA 92093 USA; 3 Division of Rheumatology, Autoimmunity and Inflammation, Department of Medicine, University of California, 9500 Gilman Drive, San Diego, La Jolla, CA 92093 USA; 4 Department of Internal Medicine, University of Nebraska Medical Center, MSB 5544, 983331, Omaha, NE 68198-3331 USA; 5 Veterans Affairs (VA) Nebraska-Western Iowa Health Care System, Omaha, NE USA

**Keywords:** Xanthine oxidase, Allopurinol, Febuxostat, Gout, Inflammation, Proteomics, Complement, TGFbeta, Inflammation, Prognostic markers

## Abstract

Urate-lowering treatment (ULT) to target with xanthine oxidase inhibitors (XOIs) paradoxically causes early increase in gouty arthritis flares. Because delayed reduction in flare burden is mechanistically unclear, we tested for ULT inflammation responsiveness markers. Unbiased proteomics analyzed blood samples (baseline, 48 weeks ULT) in two, independent ULT out trial cohorts (*n* = 19, *n* = 30). STRING-db and multivariate analyses supplemented determinations of altered proteins via Wilcoxon matched pairs signed rank testing in XOI ULT responders. Mechanistic studies characterized proteomes of cultured XOI-treated murine bone marrow macrophages (BMDMs). At 48 weeks ULT, serum urate normalized in all gout patients, and flares declined in association with significantly altered proteins (*p* < 0.05) in clustering and proteome networks in sera and peripheral blood mononuclear cells. Sera demonstrated altered complement activation and regulatory gene ontology biologic processes. In both cohorts, a treatment-emergent serum interactome included key gouty inflammation mediators (C5, IL-1B, CXCL8, IL6). Last, febuxostat treatment decreased complement activation biologic process proteins in cultured BMDMs. Reduced gout flares are linked with a XOI treatment-emergent serum protein interactome that includes inflammation regulators, associated with altered complement activation and regulatory biologic processes. Serum and leukocyte proteomics could help identify when gouty inflammatory processes begin to subside in response to ULT.

*Trial Registration*: ClinicalTrials.gov Identifier NCT02579096, posted October 19, 2015.

**Supplementary Information:**

The online version contains supplementary material available at 10.1038/s41598-024-74154-5.

## Introduction

Gout is characterized by tissue deposition of monosodium urate (MSU) crystals, which trigger acute arthritis flares that characteristically are excruciatingly painful and incapacitating^1,2^. Exogenous factors, including joint trauma, certain dietary excesses, and alcohol consumption, can trigger flares^3–5^. Gout flares are treated with nonsteroidal anti-inflammatory drugs, corticosteroids, and colchicine, which are nonselective, frequently toxic, and burdened by frequent drug-drug interactions^1,6,7^. Undertreated, gout commonly progresses to more frequent flares, chronic arthritis, and permanent joint damage^1^. Gout also is linked to prevalent comorbidities mediated by low-grade inflammation (e.g., obesity, type 2 diabetes, atherosclerosis, chronic kidney disease)^1,8^.

Pharmacologic treatment of hyperuricemia is central to gout management, with XOIs (principally allopurinol or febuxostat) the most commonly prescribed drugs^6,7^. However, effective XOI urate-lowering treatment (ULT) to target also paradoxically induces an elevated gout flare burden early in treatment^6,7,9^. Remodeling of articular MSU crystal deposits, with consequent release of free crystals, appear to mediate increased gout flares in the first year after ULT initiation^10–12^, with a variety of other mechanisms also contributing to continuing subclinical and clinically evident inflammation^13^. MSU crystals stimulate inflammation in large part by activating monocytes and macrophages, promoting NLRP3 inflammasome-mediated IL-1b release, and neutrophil influx and activation that amplify the inflammatory cascade^1,13^. C5 cleavage on the MSU crystal surface, and consequent C5b-9 complement membrane attack complex (MAC) assembly and membrane pore-forming activity mediate the inflammatory process in gouty arthritis^14,15^.

Recent clinical trials have demonstrated that effective XOI urate-lowering treatment (ULT) to target eventually reduces gout flare burden and synovitis between 1 and 2 years therapy^16–18^. Importantly, flares decrease in this time frame, despite total resolution of urate crystal deposits being far slower and very difficult to achieve^10^. In addition, systemic inflammation continues even in the periods between flares and in clinical remission^19^. In clinical practice, this situation is associated with lack of clarity on how long anti-inflammatory gout flare prophylaxis, typically using low dose colchicine, is necessary after initiating ULT and achievement of the serum urate target^9^.

XOI drugs exert anti-inflammatory effects in monocytes and some other cells, including by antioxidant and urate-lowering effects^20–24^. For example, XOI drugs inhibit NLRP3 inflammasome activation, IL-1b release, and chemokine expression in cultured monocyte/macrophage lineage cells^20,21^. In vivo, XOI drugs limit mouse models of atherosclerosis, metabolic dysfunction-associated steatohepatitis, and certain other diseases involving low-grade chronic inflammation and oxidative stress^20–24^. Hence, we conducted a seminal study to test the hypothesis that sustained, effective ULT remodels inflammatory networks in gout by 48 weeks therapy, that XOI could contribute to this effect, and that this could be detectable using unbiased proteomics.

The data revealed the ability of proteomics to detect anti-inflammatory changes in cultured XOI-treated macrophages, and in response to sustained, effective XOI-based ULT in gout patient sera and PBMCs. Our results provide unbiased evidence that sustained treat to target ULT in gout affects complement activation and other inflammatory pathways, and that XOI inhibition may contribute to remodeling of pathways that regulate gouty inflammation.

## Methods

### Subjects

As previously reported in detail^25^, Cohort 1 and Cohort 2 human subjects were studied under informed consent, and with local IRB approval (at the Jennifer Moreno San Diego Veterans Affairs Medical Center, and at the University of Nebraska Medical Center, respectively). All experiments were performed in accordance with relevant guidelines and regulations. Human subjects samples and clinical and clinical laboratory data were collected specifically in prospective study ancillary to the national, multi-site comparative effectiveness ULT trial VA CSP594 STOP GOUT, whose protocol and CONSORT statement were previously published^18^. In that trial, gout patients were randomized to a treat to urate target ULT regimen using allopurinol or the more selective XOI febuxostat. Unless contraindicated, colchicine was prescribed as the primary anti-inflammatory gout flare prophylaxis, with colchicine routinely stopped at 6 months ULT. Twenty consecutive patients meeting the 2015 ACR/EULAR gout classification criteria^26^, and with current hyperuricemia, were recruited from the Rheumatology Outpatient Clinic at the San Diego site^25^. Once again^25^, the gout validation cohort (Cohort 2, *n* = 30)) was from the University of Nebraska Medical Center, in Omaha, NE research site, under informed consent, with the same trial XOI dose escalation and limited time colchicine prophylaxis dosing protocols, and with local IRB approval. Subjects with active flare, or CRP elevated over 2 mg/L at study onset and endpoints were not excluded from analyses. We previously characterized Cohort 1 gout patient metabolomic profiles at time zero and 12 and 24 weeks of treat to target ULT, done in a blinded way for the XOI used, and following the trial protocol^25^.

### Proteomics

Sera were obtained from both cohorts, with peripheral blood mononuclear cells (PBMCs) also prepared from Cohort 1 samples. All subjects were clinically assessed by study physicians for palpable tophaceous disease and presence of active flare or quiescent arthritis, with co-morbidities and current medications also recorded.

For serum collection, research personnel collected non-fasting blood samples into 10 ml BD Vacutainer Blood Collection Tubes containing spray-coated silica and a polymer gel to facilitate serum separation. Following 30 min incubation at room temperature, tubes were centrifuged for 10 min at 2000×g and sera were transferred into 1.7 ml tubes and immediately frozen and stored at − 80 °C until analyses were performed.

For PBMC preparation, non-fasting blood samples collected into 10 ml BD Vacutainer K2 EDTA Plus Blood Collection Tubes were transferred to a conical tube containing equal volume of PBS (~ total 20 ml). The samples were then layered over Sigma Histopaque^®^-1077 (20 mL) in 50 mL conical tubes at room temperature, followed by centrifugation at 400×g in a swinging bucket centrifuge for 30 min at room temperature with no brake. The white cellular layer containing PBMCs at the interface between the plasma and density gradient was collected and washed in PBS by dilution and centrifugation for 10 min at 250×g. PBMC pellets were immediately frozen and stored at − 80 °C until analyzed.

## Mass spectrometry proteomics

Sample preparation for proteomic analyses of BMDMs and patient sera was done as we previously described in extensive detail^27^, with slight modification to the sample digestion protocol, which used 10 µg trypsin in 50mM TEAB at 47˚C for 3 h. After protein extraction and trypsin digest, 50ug aliquots of samples were reserved for TMT pro-labeling^27^. Bridge channels for downstream data analysis of serum samples, were prepped by combining 5 µg of all samples; 50 µg aliquots of our bridge sample were then prepared for each TMT-plex (5 total).

## Mass spectrometry data acquisition

Serum and BMDM proteomic data were acquired as described in detail^27^. In brief, serum and BMDM proteomic data were acquired through an Thermo Orbitrap Fusion equipped with a Thermoeasy nLC 1000. For Mass spectrometry data search, raw mass spectrometry files were searched using Proteome Discoverer 2.5.0.400. The SEQUEST algorithm was used for spectral matches of raw data with in silico generated protein databases. Serum samples were searched against the UniProt *Homo sapiens* proteome (05-06-2023) and BMDM samples were searched against the *Mus musculus* proteome (05-06-23).

## Mass spectrometry metabolomics

Sample preparation of patient sera for metabolomics were essentially as previously described^27^. In brief, for data Analysis, metabolite features were first normalized to the intensity of value of the internal standard, sulfamethazine, in each sample and then multiplied by 1E6. Missing values (with peak intensities of 0) in metabolite features were set to NA. Then, features with more than 20% missing values per group (timepoint) were removed from analysis. Missing values in remaining features were imputed using K-Nearest Neighbor (KNN) imputation using the ‘impute’ R package (1.68.0). Intensity values were then log2 transformed.

Principal coordinate analysis (PcoA) was conducted with metabolite features, using Bray-Curtis distance calculation in the ‘stats’ R package. PERMANOVA analysis was conducted using categorical metadata and metabolite features using Bray-Curtis distance calculation in the ADONIS R package. Binary comparisons between timepoints were done through the R ‘stats’ package using Students T-test. Volcano plots were created in GraphPad Prism. All other plots were made using ‘ggplot’ package in R. MetaboAnalyst (5.0) was used for metabolite functional enrichment analysis using MS peaks ranked by Student’s T test p-values. A p-value cutoff of 0.05 was used for the mummichog algorithm.

## Murine bone marrow derived macrophage (BMDM) preparation

Mouse macrophage studies were done using a protocol approved by the Jennifer Moreno San Diego Veterans Affairs Medical Center Institutional Animal Care and Use Committee (IACUC). All experiments were performed in accordance with ARRIVE guidelines and other relevant ethics and veterinary practice guidelines and regulations. No experiments were performed on live mice. To prepare mouse BMDMs for in vitro studies, 12-week-old C57BL/6 male mice (from The Jackson Lab, Bar Harbor, ME) were euthanized using the carbon dioxide (CO_2_) inhalation method, according to the 2020 American Veterinary Medical Association​ (AVMA) Guidelines for the Euthanasia of Animals. Bone marrow cells were then flushed from femur and tibia bones of these mice and were cultured in vitro in RPMI containing 10% FBS, penicillin (100 U/ml), streptomycin (100 µg/ml), and, for the source of Macrophage-Colony Stimulating Factor, 20% L929 conditioned media for 7 days. BMDMs generated from 3 individual mice (*n* = 3 biological replicates) were used for the in vitro experiments.

### Statistical analyses

Paired statistical analyses of gout patient serum and PBMC samples across two timepoints (UCSD cohort), and for three timepoints for sera (Nebraska cohort), were conducted to identify significantly altered proteins. Unpaired statistical analyses were conducted for the cultured mouse BMDM samples. Significantly altered proteins were calculated using a Wilcoxon matched pairs signed rank test using Graphpad Prism, with p-value adjusted values using Benjamini and Hochberg false discovery rate found in Supplementary Table 1.

For multivariate Analysis, Principal Component Analysis (PCA) was conducted using the ‘stats’ R package using all normalized protein features. Principal Coordinate Analysis (PCoA) was conducted using the ‘stats’ R package using the Euclidean Distance Matrix (EDM) of normalized protein features. PERMANOVA analysis was used to calculate data influence by metadata categories.

Gene Ontology enrichment analysis was conducted through input of significantly altered proteins in both diseases to their respective controls into Cytoscape. Protein interactome analysis was conducted through input of significantly altered proteins in both diseases to their respective controls into String-DB with an interaction confidence of 0.700 (high-confidence). For “pin dropping” interactome analysis, an abbreviated list of known substantial gouty inflammation mediators was appended to a list of significantly abundant proteins in either proteomics patient cohorts, and subjected to interactome analysis on STRING-DB.

## Results

### Effects of Febuxostat on BMDMs in vitro

**Fig. 1:**
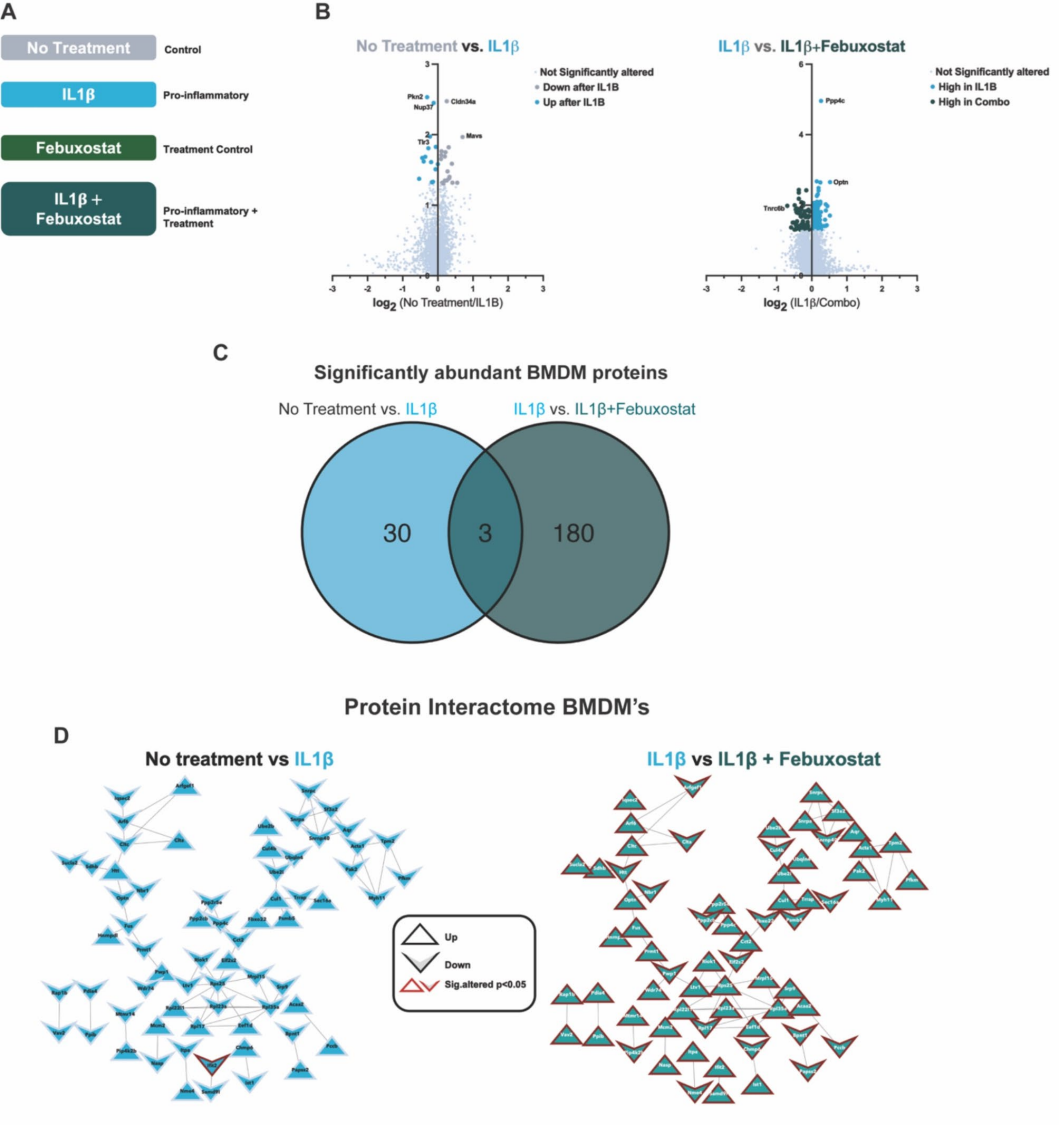
Bone Marrow Derived Macrophage (BMDMs) Proteomics. (**A**) BMDM treatment schematic. (**B**) Volcano plots of log2- fold change relative protein abundance versus log10 p-value. Points are colored by condition they are found higher in and sized by p-value significance (p-value < 0.05, Wilcoxon signed rank test). (**C**) Venn Diagram displaying overlap of differentially abundant proteins in IL1β and IL1β + Febuxostat treated macrophages. (**D**) Protein interactome from String-DB using significantly altered proteins in respective binary comparison of BMDM treatments. Nodes are shaped based on the direction of relative abundance change after respective treatments and outlined in red if found to be significantly altered (p-value < 0.05).

We incubated BMDMs with IL-1β to model the gout pro-inflammatory state (4,14) (Fig. 1A)^4,13^. Cells were treated with and without the selective XOI febuxostat, since allopurinol non-selectively inhibits both purine and pyrimidine metabolism^28^. We first identified significantly altered proteins between untreated and IL-1β-treated macrophages (mock gouty inflammation group) in vitro, with 32 proteins found to be significantly altered in response to IL-1b (Fig. 1B, left). Next, we compared IL-1β-treated macrophages with febuxostat co-treated macrophages, which demonstrated suppression of multiple pro-inflammatory proteome changes triggered by IL-1β. Specifically, we found 184 significantly altered (*p* < 0.05) proteins (Fig. 1B, right), of which 71 proteins were found to interact via STRING-DB analysis (confidence = 0.700) (Fig. 1C, right).

### Effects of XOI-based ULT to target in gout patients

#### Validation of XOI treatment effects on purine metabolism and the serum metabolome

We previously validated XOI treatment effects on purine metabolism in Cohort 1^25^. Here, we conducted untargeted metabolomics on sera of gout patients on effective serum treat to target ULT in Cohort 2 subjects treated with either febuxostat or allopurinol for 48 weeks. We annotated metabolite features using the Global Natural Products Social Molecular Networking (GNPS) platform. Since timepoint significantly influenced our paired proteomic data set, we conducted paired binary comparisons between timepoints. Comparison of baseline (BL) and proteomics endpoint 48wks of ULT revealed several significantly altered metabolites, with some significantly changed by 24wks ULT (Supplemental Fig. 2A). Functional enrichment analysis of all identified metabolite features, using MS1 peak information, validated serum metabolome changes in purine and pyrimidine metabolism in Cohort 2 in this study. These findings were associated with significant changes in multiple other pathways, including arachidonic acid metabolism, and most pronounced for linoleate metabolism at 24 and 48wks ULT (Supplemental Fig. 1B). The new findings for Cohort 2 reinforced previously published effects of XOI treatment on the serum metabolome in gout patients of Cohort 1.

### Effects of XOI treatment to urate target on the serum proteome

**Fig. 2:**
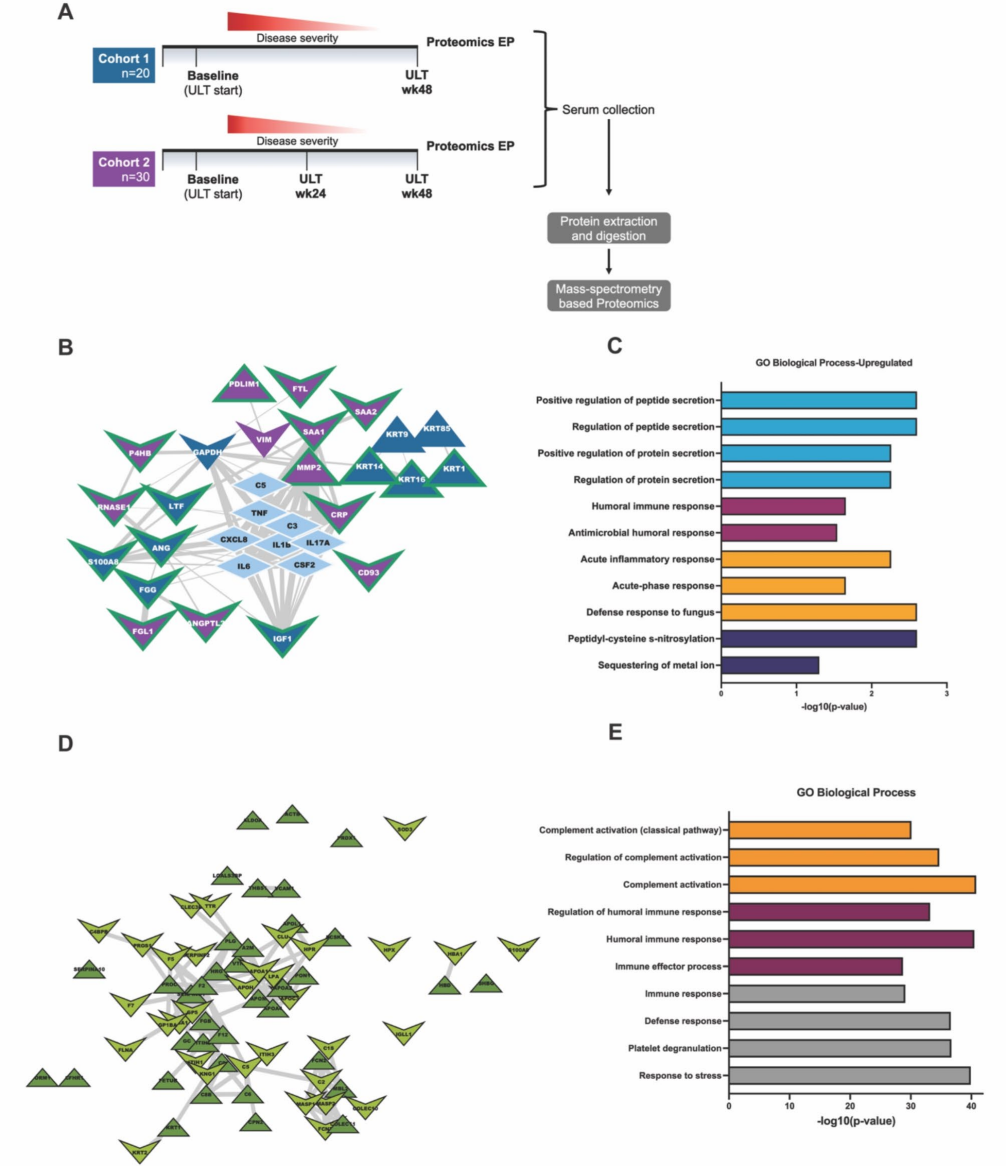
Patient Serum Proteomics. (**A**) Experimental design for proteomics studies in gout patient cohorts. Cohort 1 = UCSD Cohort, Cohort 2 = Nebraska cohort. (**B**) Protein interactome from String-DB using significantly altered proteins identified in each cohort independently along with central gout mediators. Nodes are colored by cohort they were found to be significantly altered in and shaped by their direction of change after treatment with ULT. Edges are sized by strength of interaction. (**C**) Gene ontology enrichment analysis of significantly altered proteins from both proteomic cohorts. Enrichment was conducted on Cytoscape with the Human Proteome as background. (**D**) Protein interactome of the detected overlapping proteins (nodes colored in green) that changed in the same direction in both cohorts after 48wks of ULT. Shape of the nodes is based on respective direction of change after ULT. (**E**) Gene ontology enrichment analysis of overlapping proteins from both cohorts. Enrichment was conducted on Cytoscape with the Human Proteome as background.

We performed quantitative proteomic analysis on patient serum samples to understand global serum proteome changes before and at 48wks XOI-based ULT. Experimental approach, patient demographics and changes in serum urate are summarized (Fig. 2A, Supplemental Fig. 1). Briefly, patient racial and ethnic backgrounds varied, with Cohort 1 patients identified as largely White and Black, and Cohort 2 identified as predominantly White (Supplemental Fig. 1D and 1 H). Additionally, we observed overall decrease in serum urate levels after 48wks ULT and patient reported flares, but relatively stable C-reactive protein (CRP) levels after ULT in both cohorts (Supplemental Fig. 1A-C & 1E-G).

Examining each cohort independently from Baseline (BL) to serum proteomics Endpoint (48 wks of ULT; EP), we found 24 and 32 significantly altered proteins (*p* < 0.05, Wilcoxon signed-ranks test) for Cohort 1 and 2, respectively. To gain functional information and elucidate the potential roles of significantly altered proteins in gouty inflammation we performed interactome analysis through STRING-db. This was accomplished by “pin-dropping” established gouty-inflammation mediators, known to be below the mass spectrometry detection limits^29^, along with the significantly altered proteins from both cohorts. We identified 23 high confidence interacting proteins in Cohorts 1 and 2 (Fig. 2B), which showed alterations in networked inflammation mediators not detectable by conventional serum biomarker profiling, including PPBP/CXCL7, KRT16, TGFB1, and IGF-I. These novel biomarkers of XOI treatment effects were identified within an interactome with central gout mediators including IL-1B, CXCL8, IL6, and C5 (Fig. 2B). Gene Ontology enrichment analyses revealed to belong to 4 major categories: Innate immune response, humoral immune response, protein/peptide secretion, and post-translational modification of proteins (Fig. 2C).

We next sought to understand overlapping changes between our two independently sampled patient cohorts. First, we analyzed proteins identified in both our cohorts by stratification to include only those proteins that changed in the same direction in both cohorts over the course of ULT treatment (baseline to proteomics endpoint). There were 277 overlapping protein identifications between both independent cohorts. In the interactome analysis, we observed 135 high confidence interacting proteins (Supplemental Tables 1 & Fig. 2D). Moreover, we identified 70 proteins that were similarly altered at 48wks ULT (Supplemental Tables 1 & Fig. 2D) in both cohorts. Analysis of the 70 proteins similarly altered in association with ULT in both cohorts (Fig. 2D) showed enrichment in both innate or humoral immunity gene ontology biological process categories (Fig. 2E). These included classical pathway activation, overall complement pathway activation, and regulation of complement pathway activation (Fig. 2E).

#### XOI treatment to serum urate target effects on the PBMC Proteome

**Fig. 3:**
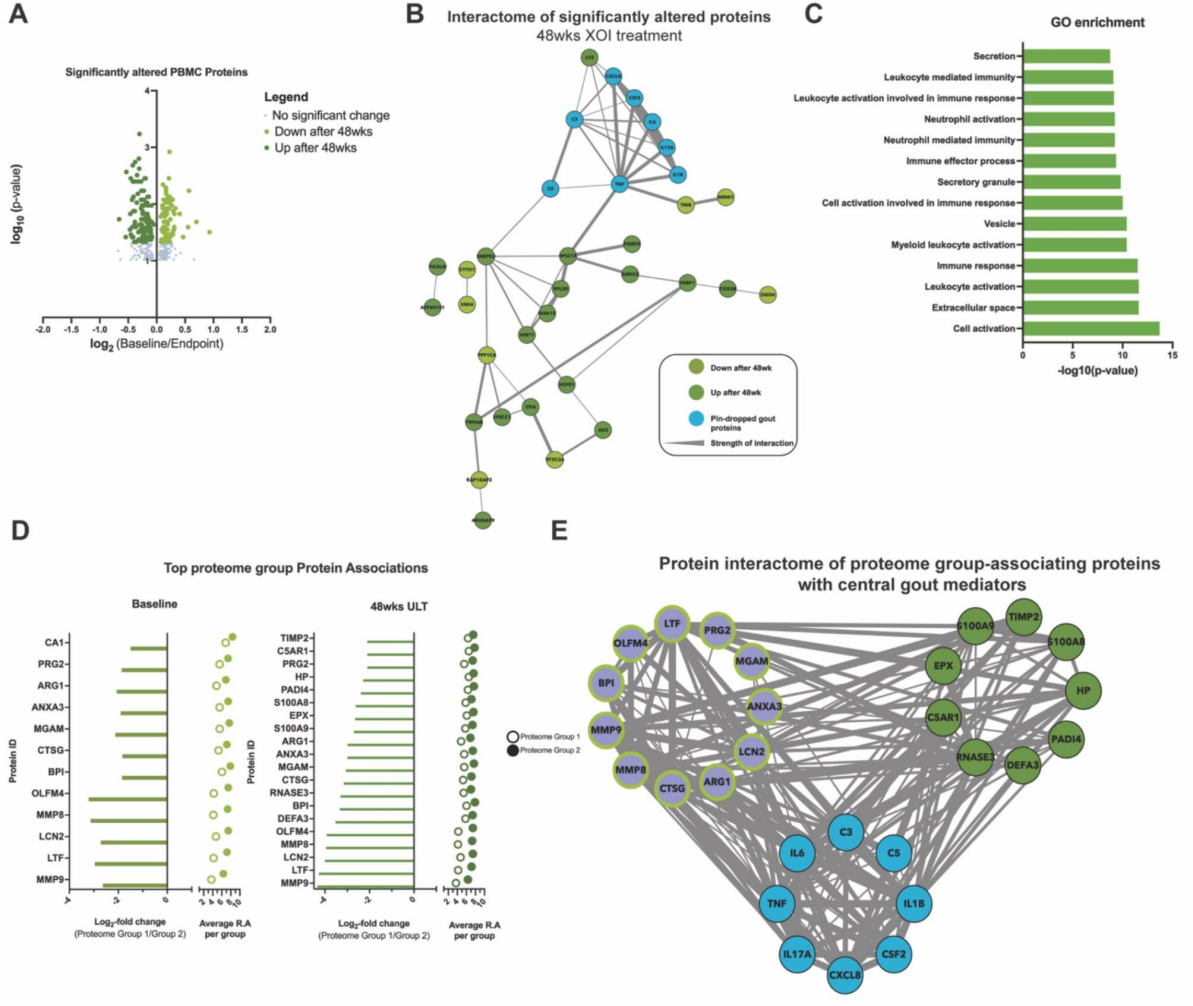
PBMC proteomics. (**A**) Volcano plots of log2- fold change relative protein abundance versus log10 p-value. Points are colored by condition they are found higher in, and sized by p-value significance (p-value < 0.05, Wilcoxon signed rank test). (**B**)Protein interactome from String-DB using significantly altered proteins after ULT treatment of gout patients. Nodes are colored by group they are found to have higher relative abundance. (**C**)Gene ontology enrichment analysis of significantly altered proteins after ULT. Enrichment was conducted on Cytoscape with the Human Proteome as background. (**D**)PBMC patient proteome-associated protein abundances to understand PBMC patient proteome separation conducted at baseline and proteomics endpoint (48wks). (**E**)Protein interactome from String-DB using top protein drivers of PBMC patient proteome separation along with “pin-dropped” central gout mediators. Nodes are colored by group they are found to have higher relative abundance.

To further characterize in vivo response to XOI-based ULT in gout, we isolated PBMCs from Cohort 1 patients. We identified 197 significantly altered proteins at 48wks ULT (*p* < 0.05, Fig. 3A), with 42 high-confidence (> 0.700) interacting proteins (Fig. 3B). Gene enrichment analysis found these proteins belonging largely to secretion, leukocyte, and neutrophil activation gene ontology pathways (Fig. 3C). Moreover, the KRT protein findings for serum proteins were further validated in the PBMC proteomics studies, as shown by their presence as significantly altered proteins in the PBMC proteomics.

We next sought to understand how patient metadata associated to the PBMC proteome. To accomplish this, we first performed a metadata association analysis (Supplemental Fig. 2A) followed by correlation analysis between patient samples. Metadata association analysis through PERMANOVA analysis identified no significant influences from sample metadata categories, such as serum urate or CRP levels, or cytokine levels from IL1B, IL6, and IL8 (Supplemental Fig. 2A). Spearman rank correlation analysis of patient PBMC proteome samples identified two distinct proteome groups (Supplemental Fig. 2A-B). To study proteomics features that drove this patient separation, we performed PERMANOVA and statistical analysis between both proteome groups 1(*n* = 5) and 2 (*n* = 14). We analyzed samples separated by timepoint and identified the top scored proteins at Baseline and 48wks of ULT (Fig. 3D). We identified overlapping protein drivers of separation at both timepoints, and interactome analysis of identified driver proteins at both timepoints along with “pin-dropped” gout proteins (Fig. 3E) found strong and high confidence (> 0.700) interactions between known gout mediators and top identified proteins, particularly MMP9 and other proteins identified at 48wks ULT. Hence, PBMC proteome analysis further teased apart XOI-based ULT effects in gout patients while highlighting anti-inflammatory effects.

## Discussion

Gout requires a unique approach to arthritis targets and biomarkers of the response to XOI-based ULT, due to variable phenotypes, and weaving of urate homeostasis, comorbidities, and inflammatory arthritis^1–5,8^. In contrast to the genetics of urate biology, genome-wide association studies have identified few genetic coding variants potentially involved in gouty arthritis^30,31^. Therefore, this biomarker study profiled the serum proteome of gout patient sera at 48wks sustained ULT to urate target, here using XOI, and with achievement of reduced flare burden and serum urate in two independent cohorts.

Findings for serum proteomics gene ontology biologic processes at 48wks XOI-based treat to target ULT, in both cohorts studied, included altered classical and overall complement pathway activation and complement regulatory biology processes. Complement activation, including C5b-9 MAC assembly and activity in combination with C5a generation, contributes substantially to the inflammatory process in model gouty arthritis^14,15,32^. XOI-based ULT to serum urate target was associated with increase in serum of TGFB1, which promotes model gout flare resolution by suppressing macrophage activation by crystals^33^. Conversely, IGF-I, which cross-talks with and can synergize with TGF-beta, was decreased in serum at 48wks ULT^34^. We detected decreased lactoferrin (LTF, also known as lactotransferrin), a neutrophil-released co-activator of the lubricin-degrading serine protease Cathepsin G^35^. Importantly, Cathepsin G is a major degrader of lubricin, which functions as a substantial constitutive suppressor of gouty inflammation and urate production by synovial resident macrophages^36^ We also observed an increase in monocyte/macrophage-expressed keratin-related proteins (KRT9,14,16), further validated by Cohort 1 gout patient PBMC proteomics. KRT16 is implicated in monocyte to macrophage differentiation, and MMP-1 and innate immune responses to tissue damage in epithelia^37^. Last, STRING-db analyses of significantly altered proteins from both cohorts revealed that the serum protein interactome network altered by XOI-based ULT encompassed a core group of central mediators of gouty inflammation (including IL-1B, CXCL8, IL6, C5)^4^.

Robustness of our findings on effects of effective ULT on the serum protein interactome discovered here was buttressed by a group of parallel studies. First, in this context, previously published evidence in gout Cohort 1 that the ULT regimen altered the serum metabolome, and the serum lipidome in gout Cohorts 1 and 2, and effects of febuxostat on lipolysis in cultured adipocytes^25^. Moreover, the current study demonstrated that the serum metabolome was significantly altered for purine and pyrimidine metabolism in Cohort 2, associated with significant changes in multiple other pathways, most pronounced for linoleate metabolism at both 24wks and 48wks ULT. Second, analyses of the Cohort 1 proteome of gout patient PBMCs identified 42 high-confidence interacting proteins belonging largely to secretion, leukocyte, and neutrophil activation gene ontology pathways. The KRT findings for serum proteins were validated in the PBMC proteome. In addition, we found strong and high confidence (> 0.700) interactions between known gout mediators and EFS identified proteins, particularly in the proteins identified at 48wks of ULT, including MMP9. By comparison, no significant difference in MMP9 abundance levels was identified between BL and 48wks of ULT, further study would be needed to validate significance of differences between PBMC proteome groups 1 and 2. The collective results of PBMC proteome analysis further teased apart the effects of XOI-based ULT in gout, and highlighted anti-inflammatory effects of XOI-based ULT on these leukocytes as a whole.

We employed in vitro studies that characterized effects of the selective XOI febuxostat on the proteome of cultured murine BMDMs stimulated by the major gouty inflammation driver IL-1b. Febuxostat suppressed multiple pro-inflammatory IL-1b-induced changes in the macrophage proteome. Analyses of gene ontology enrichment of proteins found in the macrophage protein interactome revealed that in vitro XOI treatment of activated BMDMs broadly reversed many pro-inflammatory responses. Notably, the most pronounced pathway changes were seen in classical and alternative pathway complement activation. This result reinforced the evidence for XOI-treatment effects on complement activation and complement regulatory gene ontology biologic processes in the gout patient serum proteome. Febuxostat also altered lymphocyte-mediated immunity, fibrinolysis, and cytolysis gene ontology pathways in cultured macrophages in response to IL-1b. Our findings in cultured macrophages and gout patient PBMCs were novel partly because previous studies have suggested that both hyperuricemia and urate crystals program elevated monocyte inflammatory responses in vitro and that hyperuricemia primes model gout inflammation in mice in vivo model gout^38–40^.

A pro-inflammatory serum proteome signature was recently characterized in asymptomatic hyperuricemia (AH) by targeted proteomics^41^. The approach used the Olink Target 96 Inflammation Panel™^41^, distinct from the unbiased mass spectrometry-based approach utilized in the current study. The methodology employed dual recognition by oligonucleotide-labelled antibody probe pairs and DNA-coupled quantitative PCR, designed to detect specific immunoregulatory proteins below mass spectrometry detection limits^41^. Upregulated serum immunoregulatory proteins in AH group included the mTOR effector 4E-BP1, IL-18R1, multiple growth factors, chemokines, and members of the IL-6 cytokine and TNF superfamily^44^. A Th17 cell signature, and increases in inflammation-dampening IL-10 and FGF21 also were identified^44^. Using the same targeted serum proteomics approach, a small sub-study of 13 subjects before and 3 months into successful XOI-based treat to target ULT revealed significant downregulation of LIF-R. CDCP1, IL-18, NT-3, IL10RB, CCL28, CCL11, and SLAMF1^44^.

A second, recent study of the serum proteome in gout flare, again using the Olink targeted proteomics approach in two independent cohorts, identified four markers elevated during gout flare compared to the treat to target phase and in-between flare (intercritical) phase. These inflammation-mediating proteins were tumor necrosis factor superfamily 14 (TNFSF14), IL-6, colony-stimulating factor 1 and vascular endothelial growth factor A^42^.

The differentially detected proteins in both these referenced targeted proteomics studies^44,42^ were predominantly cytokines and growth factors below the detection limits of our unbiased mass spectrometry serum proteomics approach. Therefore, the design, molecular proteomics approach, and sample size of the current study were unique and provided distinct information on the effects of XOI-based ULT treatment on inflammation mechanisms in gout. Nonetheless, factors that can modulate and perpetuate systemic gouty inflammation after ULT have been identified by approaches other than proteomics. Such factors include changes in a subset of CD14 positive monocytes, overactivation of CD8 + T cells, and upregulated arachidonate metabolism^19^.

Hyperuricemia increases blood monocyte population expansion in vivo in humans^39^, however, monocytes, and other mononuclear leukocytes, are heterogeneous, and can be recruited into diseased or challenged tissues. As such, one limitation in this study is that monocytes are normally only a small fraction (i.e., *≤* 10%) of PBMCs^43^. PBMCs, though a source of highly informative biomarkers for inflammatory diseases, are highly heterogeneous^44^. That underlines a limitation of this study, in that PBMCs only were obtained at the Cohort 1 site.

This trial did not have a placebo group. Moreover, the clinical trial did not include a uricosuric treatment arm, with the infrequently employed and frequently contraindicated USA-approved drug probenecid, to isolate effects due to serum urate-lowering without XOI. We did not exclude subjects with flare at onset of study enrollment and first and final blood sampling, or CRP higher than 2 mg/L. However, such CRP elevation was present at blood sampling in less than a handful of subjects. Also, we did not study gout patient controls, from the same clinical trial, who failed to achieve serum urate target. That said, the proportion of such subjects overall in the VA STOP GOUT trial was low (i.e., ~ 20%)^18^, and all those subjects were considered at least partially treated since they received XOI-based ULT.

In conclusion, a novel, functionally important network of physically interacting proteins in gouty inflammation emerged in association with response to sustained XOI-based ULT that effectively reduced gout flare burden. Potential clinical significance of the results, especially for data from the clinical trial, included that the treat to target ULT regimen is associated with early increase in flare activity before gout flares eventually decrease^9^. Moreover, the current study provides further support for the use of serum proteomics, including biomarker approaches highlighting the complement pathway and the inflammatory secretome, to help identify responsiveness of gouty inflammation to ULT pharmacotherapy, and for characterization and prognosis of different clinical phenotypes in gout^36,41,45^.

## Electronic supplementary material

Below is the link to the electronic supplementary material.

Supplementary Material 1

Supplementary Material 2

## Data Availability

Raw proteomic and metabolomic data, as well as protein abundance tables can be accessed through massive.ucsd.edu via a MSV identifiers MSV000093638 (BMDMs) and MSV000093652 (Patient Serum).

## Abbreviations

AH: Asymptomatic hyperuricemia
BMDM: Bone marrow-derived macrophage
CDCP1: CUB domain-containing protein 1
EFS: Ensemble Feature Selection
FGF21: Fibroblast growth factor 21
IL10RB: IL-10 Receptor subunit beta
KRT: Keratin
LIF-R: Leukemia Inhibitory Factor Receptor
LTF: Lactoferrin
MMP: Matrix metalloproteinase
MSU: Monosodium urate
NT-3: Neurotrophin 3
NLRP3: NLR Family Pyrin Domain Containing 3
PCA: Principal Component Analysis
PCoA: Principal Coordinate Analysis
PBMC: Peripheral blood mononuclear cell
SLAMF1: Signaling Lymphocytic Activation Molecule Family Member 1
TMT: Tandem mass tag
ULT: Urate lowering therapy
XOI: Xanthine Oxidase Inhibition

## References

[1] Dalbeth, N., Merriman, T. R. & Stamp, L. K. Gout. *Lancet* **388**, 2039–2052. 10.1016/s0140-6736(16)00346-9 (2016). 27112094 10.1016/S0140-6736(16)00346-9

[2] Teoh, N. *et al.* The challenges of gout flare reporting: mapping flares during a randomized controlled trial. *BMC Rheumatology* **3**. 10.1186/s41927-019-0075-6 (2019). 10.1186/s41927-019-0075-6 PMC6615178 31334482

[3] Zhang, Y. et al. Purine-rich foods intake and recurrent gout attacks. *Ann. Rheum. Dis.* **71**, 1448–1453. 10.1136/annrheumdis-2011-201215 (2012). 22648933 10.1136/annrheumdis-2011-201215 PMC3889483

[4] Terkeltaub, R. What makes gouty inflammation so variable? *BMC Medicine* **15**. 10.1186/s12916-017-0922-5 (2017). 10.1186/s12916-017-0922-5 PMC5561591 28818081

[5] Danve, A., Sehra, S. T. & Neogi, T. Role of diet in hyperuricemia and gout. *Best Pract. Res. Clin. Rheumatol.* **35**, 101723. 10.1016/j.berh.2021.101723 (2021). 34802900 10.1016/j.berh.2021.101723 PMC8678356

[6] Fitzgerald, J. D. et al. 2020 American College of Rheumatology Guideline for the Management of Gout. *Arthritis Care Res.* **72**, 744–760. 10.1002/acr.24180 (2020). 10.1002/acr.24180 PMC10563586 32391934

[7] Richette, P. et al. 2016 updated EULAR evidence-based recommendations for the management of gout. *Ann. Rheum. Dis.* **76**, 29–42. 10.1136/annrheumdis-2016-209707 (2017). 27457514 10.1136/annrheumdis-2016-209707

[8] Choi, H. K., Mccormick, N. & Yokose, C. Excess comorbidities in gout: the causal paradigm and pleiotropic approaches to care. *Nat. Rev. Rheumatol.* **18**, 97–111. 10.1038/s41584-021-00725-9 (2022). 34921301 10.1038/s41584-021-00725-9

[9] Stamp, L. et al. Is colchicine prophylaxis required with start-low go-slow allopurinol dose escalation in gout? A non-inferiority randomised double-blind placebo-controlled trial. *Ann. Rheum. Dis.* **82**, 1626–1634. 10.1136/ard-2023-224731 (2023). 37652661 10.1136/ard-2023-224731

[10] Peiteado, D., Villalba, A., Martín-Mola, E. & de Miguel, E. Reduction but not disappearance of Doppler signal after two years of treatment for gout. Do we need a more intensive treatment? 25898174

[11] Hammer, H. B. et al. Ultrasound shows rapid reduction of crystal depositions during a treat-to-target approach in gout patients: 12-month results from the NOR-Gout study. *Ann. Rheum. Dis.* **79**, 1500–1505. 10.1136/annrheumdis-2020-217392 (2020). 32669301 10.1136/annrheumdis-2020-217392

[12] Uhlig, T. et al. Two-year reduction of dual-energy CT urate depositions during a treat-to-target strategy in gout in the NOR-Gout longitudinal study. *Rheumatology* **61**, SI81–SI85. 10.1093/rheumatology/keab533 (2022). 34247224 10.1093/rheumatology/keab533 PMC9015021

[13] Schlesinger, N., Pillinger, M. H., Simon, L. S. & Lipsky, P. E. Interleukin-1β inhibitors for the management of acute gout flares: a systematic literature review. *Arthritis Res. Ther.* **25**. 10.1186/s13075-023-03098-4 (2023). 10.1186/s13075-023-03098-4 PMC10367374 37491293

[14] Tramontini, N., Huber, C., Liu-Bryan, R., Terkeltaub, R. A. & Kilgore, K. S. Central role of complement membrane attack complex in monosodium urate crystal–induced neutrophilic rabbit knee synovitis. *Arthritis Rheum.* **50**, 2633–2639. 10.1002/art.20386 (2004). 15334478 10.1002/art.20386

[15] Russell, I. J., Mansen, C., Kolb, L. M. & Kolb, W. P. Activation of the fifth component of human complement (C5) induced by monosodium urate crystals: C5 convertase assembly on the crystal surface. *Clin. Immunol. Immunopathol.* **24**, 239–250. 10.1016/0090-1229(82)90235-5 (1982). 6749358 10.1016/0090-1229(82)90235-5

[16] Doherty, M. et al. Efficacy and cost-effectiveness of nurse-led care involving education and engagement of patients and a treat-to-target urate-lowering strategy versus usual care for gout: a randomised controlled trial. *Lancet* **392**, 1403–1412. 10.1016/s0140-6736(18)32158-5 (2018). 30343856 10.1016/S0140-6736(18)32158-5 PMC6196879

[17] Dalbeth, N. et al. Effects of febuxostat in early gout. *Arthritis Rheumatol.* **69**, 2386–2395. 10.1002/art.40233 (2017). 28975718 10.1002/art.40233 PMC5725733

[18] O’Dell, J. R. *et al.* Comparative Effectiveness of Allopurinol and Febuxostat in Gout Management. *NEJM Evidence* **1**. 10.1056/evidoa2100028 (2022). 10.1056/evidoa2100028 PMC9012032 35434725

[19] Gu, H. et al. MSU crystal deposition contributes to inflammation and immune responses in gout remission. *Cell Rep.* **42**, 113139. 10.1016/j.celrep.2023.113139 (2023). 37756161 10.1016/j.celrep.2023.113139

[20] Ives, A. et al. Xanthine oxidoreductase regulates macrophage IL1β secretion upon NLRP3 inflammasome activation. *Nat. Commun.* **6**, 6555. 10.1038/ncomms7555 (2015). 25800347 10.1038/ncomms7555 PMC4382995

[21] Nomura, J. *et al.* Xanthine oxidase inhibition by febuxostat attenuates experimental atherosclerosis in mice. *Sci. Rep.* **4**. 10.1038/srep04554 (2014). 10.1038/srep04554 PMC3971401 24686534

[22] Nomura, J. et al. Febuxostat, an Inhibitor of Xanthine Oxidase, Suppresses Lipopolysaccharide-Induced MCP-1 Production via MAPK Phosphatase-1-Mediated Inactivation of JNK. *PLoS ONE* **8**, e75527. 10.1371/journal.pone.0075527 (2013). 24086554 10.1371/journal.pone.0075527 PMC3783396

[23] Kusano, T. *et al.* Targeted knock-in mice expressing the oxidase-fixed form of xanthine oxidoreductase favor tumor growth. *Nat. Commun.* **10**. 10.1038/s41467-019-12565-z (2019). 10.1038/s41467-019-12565-z PMC6817904 31659168

[24] Nishikawa, T. *et al.* Xanthine oxidase inhibition attenuates insulin resistance and diet-induced steatohepatitis in mice. *Scientific Reports* **10**. 10.1038/s41598-020-57784-3 (2020). 10.1038/s41598-020-57784-3 PMC6972756 31965018

[25] Guma, M. *et al.* Xanthine oxidase inhibitor urate-lowering therapy titration to target decreases serum free fatty acids in gout and suppresses lipolysis by adipocytes. *Arthritis Res. Ther.* **24**. 10.1186/s13075-022-02852-4 (2022). 10.1186/s13075-022-02852-4 PMC9310412 35879786

[26] Neogi, T. et al. 2015 Gout classification criteria: an American College of Rheumatology/European League Against Rheumatism collaborative initiative. *Ann. Rheumatic Dis.* **74**, 1789–1798. 10.1136/annrheumdis-2015-208237 (2015). 10.1136/annrheumdis-2015-208237 PMC4602275 26359487

[27] Campeau, A. et al. Multi-omics of human plasma reveals molecular features of dysregulated inflammation and accelerated aging in schizophrenia. *Mol. Psychiatry* **27**, 1217–1225. 10.1038/s41380-021-01339-z (2022). 34741130 10.1038/s41380-021-01339-z PMC9054664

[28] Terkeltaub, R. Emerging urate-lowering drugs and pharmacologic treatment strategies for gout: A narrative review. *Drugs* **83**, 1501–1521. 10.1007/s40265-023-01944-y (2023). 37819612 10.1007/s40265-023-01944-y

[29] Wozniak, J. M. et al. Mortality risk profiling of staphylococcus aureus bacteremia by multi-omic serum analysis reveals early predictive and pathogenic signatures. *Cell* **182**, 1311-1327.e1314. 10.1016/j.cell.2020.07.040 (2020). 32888495 10.1016/j.cell.2020.07.040 PMC7494005

[30] Chang, W.-C. *et al.* Genetic variants of PPAR-gamma coactivator 1B augment NLRP3-mediated inflammation in gouty arthritis. *Rheumatology*, kew337. 10.1093/rheumatology/kew337 (2016). 10.1093/rheumatology/kew337 28394398

[31] Mckinney, C. *et al.* Multiplicative interaction of functional inflammasome genetic variants in determining the risk of gout. *Arthritis Res. Therapy* **17**. 10.1186/s13075-015-0802-3 (2015). 10.1186/s13075-015-0802-3 PMC4604627 26462562

[32] Cumpelik, A., Ankli, B., Zecher, D. & Schifferli, J. A. Neutrophil microvesicles resolve gout by inhibiting C5a-mediated priming of the inflammasome. *Ann. Rheumatic Dis.* **75**, 1236–1245. 10.1136/annrheumdis-2015-207338 (2016). 10.1136/annrheumdis-2015-207338 PMC4893114 26245757

[33] Steiger, S. & Harper, J. L. Neutrophil cannibalism triggers transforming growth factor β1 production and self regulation of neutrophil inflammatory function in monosodium urate monohydrate crystal–induced inflammation in mice. *Arthritis Rheumatism* **65**, 815–823. 10.1002/art.37822 (2013). 23280587 10.1002/art.37822

[34] Danielpour, D. & Song, K. Cross-talk between IGF-I and TGF-β signaling pathways. *Cytokine Growth Factor Rev.* **17**, 59–74. 10.1016/j.cytogfr.2005.09.007 (2006). 16297654 10.1016/j.cytogfr.2005.09.007

[35] Eipper, S. et al. Lactoferrin is an allosteric enhancer of the proteolytic activity of cathepsin G. *PLOS ONE* **11**, e0151509. 10.1371/journal.pone.0151509 (2016). 26986619 10.1371/journal.pone.0151509 PMC4795699

[36] Elsaid, K. et al. Amplification of Inflammation by Lubricin Deficiency Implicated in Incident, Erosive Gout Independent of Hyperuricemia. *Arthritis Rheumatol.* **75**, 794–805. 10.1002/art.42413 (2023). 36457235 10.1002/art.42413 PMC10191887

[37] Medina, A., Brown, E., Carr, N. & Ghahary, A. Circulating monocytes have the capacity to be transdifferentiated into keratinocyte-like cells. *Wound Repair Regener.* **17**, 268–277. 10.1111/j.1524-475x.2009.00457.x (2009). 10.1111/j.1524-475X.2009.00457.x 19320896

[38] Mulder, W. J. M., Ochando, J., Joosten, L. A. B., Fayad, Z. A. & Netea, M. G. Therapeutic targeting of trained immunity. *Nat. Rev. Drug Discov.* **18**, 553–566. 10.1038/s41573-019-0025-4 (2019). 30967658 10.1038/s41573-019-0025-4 PMC7069501

[39] Grainger, R., Mclaughlin, R. J., Harrison, A. A. & Harper, J. L. Hyperuricaemia elevates circulating CCL2 levels and primes monocyte trafficking in subjects with inter-critical gout. *Rheumatology* **52**, 1018–1021. 10.1093/rheumatology/kes326 (2013). 23204548 10.1093/rheumatology/kes326

[40] Crişan, T. O. et al. Uric acid priming in human monocytes is driven by the AKT–PRAS40 autophagy pathway. *Proc. Natl. Acad. Sci.* **114**, 5485–5490. 10.1073/pnas.1620910114 (2017). 28484006 10.1073/pnas.1620910114 PMC5448210

[41] Cabău, G. et al. Hyperuricemia remodels the serum proteome toward a higher inflammatory state. *iScience* **26**, 107909. 10.1016/j.isci.2023.107909 (2023). 37810213 10.1016/j.isci.2023.107909 PMC10550725

[42] Ea, H.-K. *et al.* Systemic inflammatory cytokine profiles in patients with gout during flare, intercritical and treat-to-target phases: TNFSF14 as new biomarker. *Ann. Rheumatic Dis.* ard-2023-225305. 10.1136/ard-2023-225305 (2024). 10.1136/ard-2023-225305 38373842

[43] Hamers, A. A. J. et al. Human monocyte heterogeneity as revealed by high-dimensional mass cytometry. *Arteriosclerosis Thrombosis Vasc. Biol.* **39**, 25–36. 10.1161/atvbaha.118.311022 (2019). 10.1161/ATVBAHA.118.311022 PMC6697379 30580568

[44] Haynes, L. Immunological heterogeneity. *Innov. Aging* **4**, 855–855. 10.1093/geroni/igaa057.3146 (2020).

[45] Chen, G. *et al.* Quantitative proteomics by iTRAQ-PRM based reveals the new characterization for gout. *Proteome Sci.* **19**. 10.1186/s12953-021-00180-0 (2021). 10.1186/s12953-021-00180-0 PMC8507311 34635120

