# Supplementary material for: Effective xanthine oxidase inhibitor urate lowering therapy in gout is linked to an emergent serum protein interactome of complement and inflammation modulators
Source: Sci Rep. 2024 Oct 19;14:24598. doi: 10.1038/s41598-024-74154-5 (PMC11490615; doi:10.1038/s41598-024-74154-5)
Supplement: Supplementary file 1 — Supplementary Material 1 [file 41598_2024_74154_MOESM1_ESM.docx]

Supplemental Figure 1


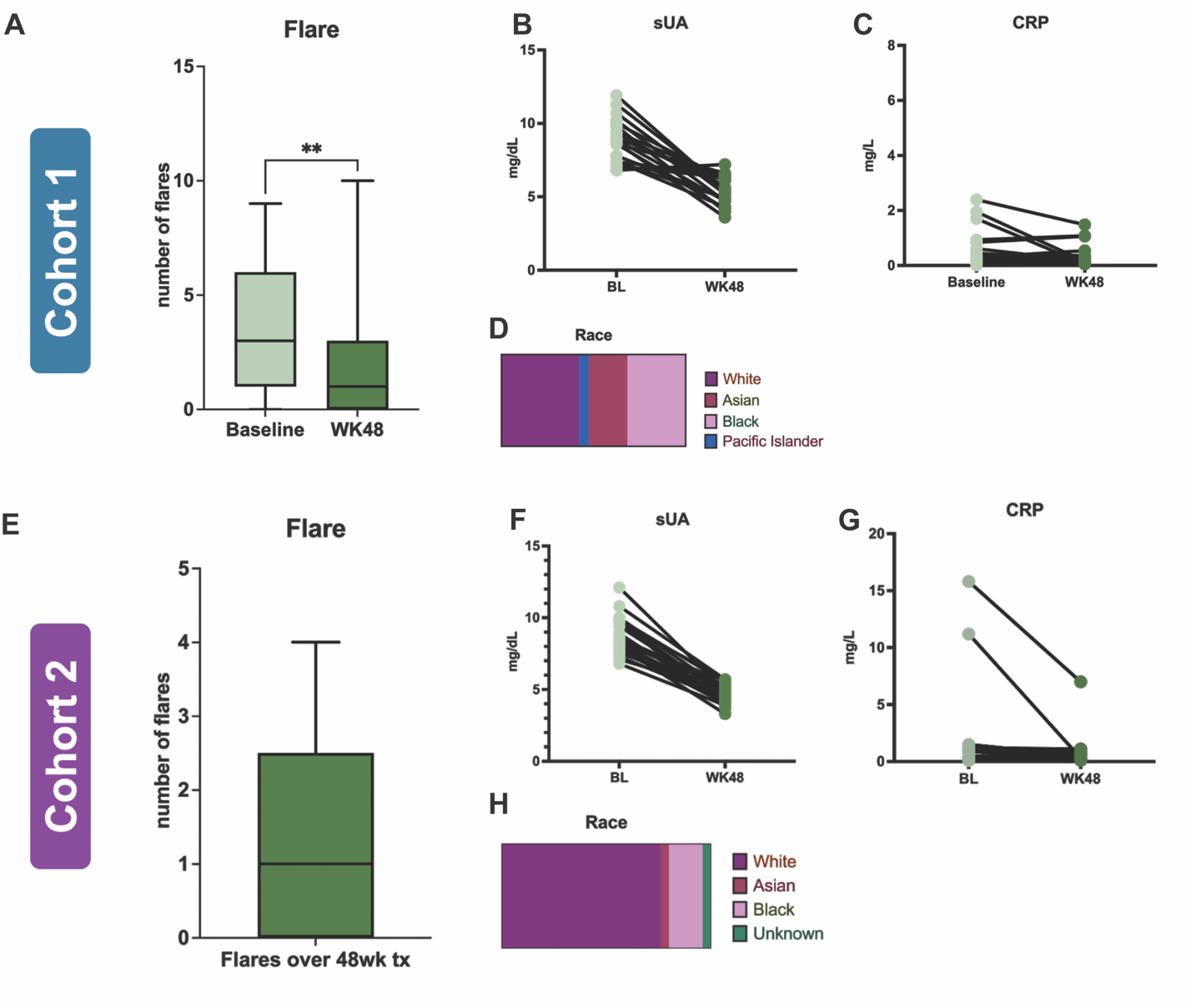


**Supplemental Figure 1.** Patient demographics for serum proteomics studies. A) Patient flare status demographics of serum proteomics Cohort 1 (UCSD). B)Patient serum urate (sUA) levels of serum proteomics Cohort 1 (UCSD). C)Patient c-reactive protein (CRP) levels of serum proteomics Cohort 1 (UCSD). D)Patient race/ethnicity demographics of serum proteomics Cohort 1 (UCSD). E)Patient flare status demographics of serum proteomics Cohort 2 (Nebraska). F) Patient serum urate (sUA) levels of serum proteomics Cohort 2 (Nebraska). G) Patient c-reactive protein (CRP) levels of serum proteomics Cohort 2(Nebraska). H) Patient race/ethnicity demographics of serum proteomics Cohort 2 (Nebraska).

Supplemental Figure 2


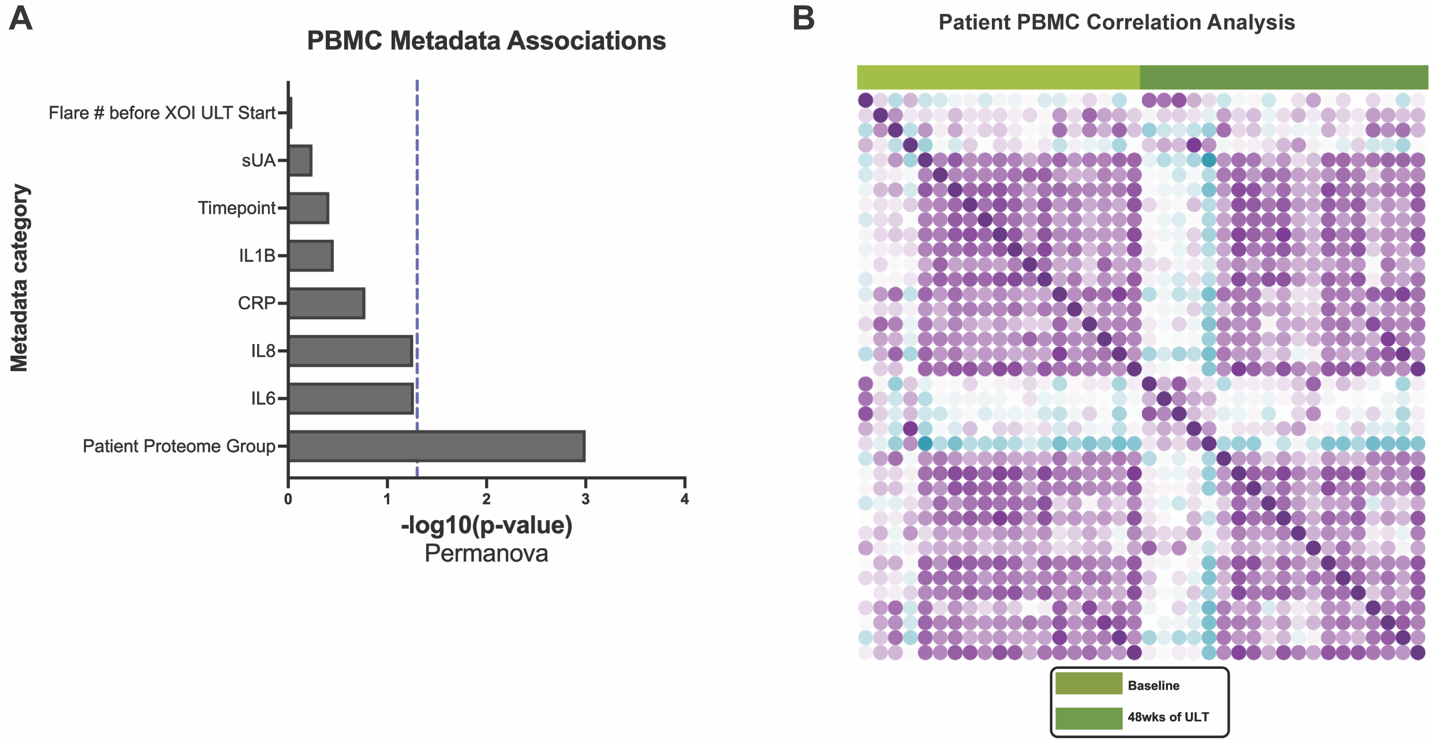


**Supplemental Figure 2.** Patient demographics for serum proteomics studies. A) Metadata associations with PBMC proteome. Dotted line displays PERMANOVA significance cutoff (p<0.05, -log_10_p-value>1.3). B) Patient PBMC Spearman correlation, organized by time point.

Supplemental Figure 3


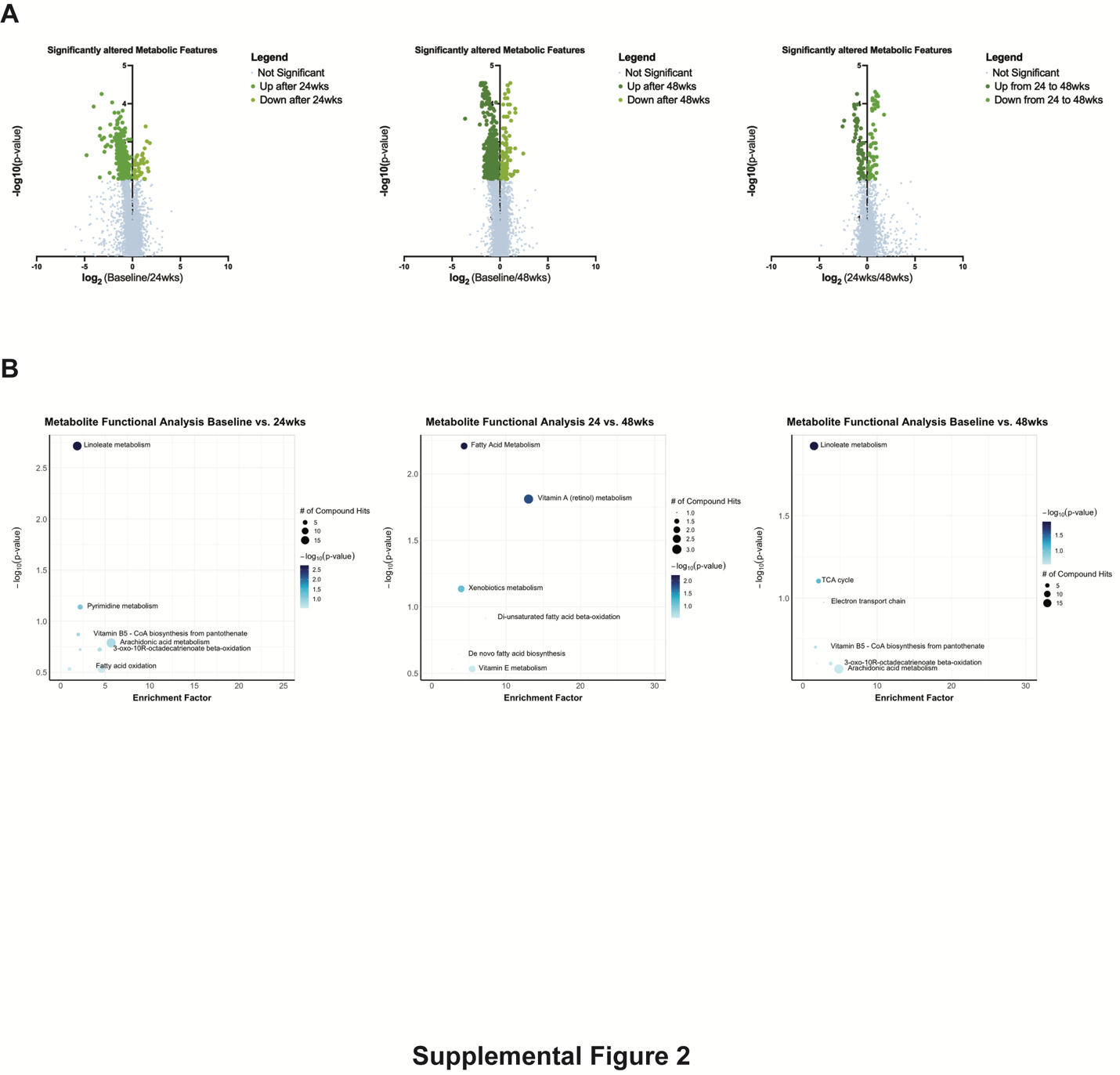


**Supplemental Figure 3.** Serum Metabolome of Cohort 2 (Nebraska). A) Volcano plots of log2- fold change metabolite abundance versus log10 p-value. Points are colored by condition they are found higher in and sized by p-value significance (p-value<0.05, Wilcoxon signed rank test). B) Metabolomics functional pathway enrichment analysis was from MetaboAnalyst 4.0 using all identified metabolite features and their respective p-value (Wilcoxon signed rank test) changes after 48wks of ULT.
